# Supplementary material for: Belief in science-related conspiracy theories is not just a matter of knowledge: The democratic quality of countries as a protective factor
Source: PLoS One. 2025 Dec 18;20(12):e0338384. doi: 10.1371/journal.pone.0338384 (PMC12714214; doi:10.1371/journal.pone.0338384)
Supplement: S1 File — (PDF) [file pone.0338384.s001.pdf]

Supplementary Information for “Belief in science-related conspiracy theories is not just a matter of knowledge: the democratic quality of countries as a protective factor”

Irene López-Navarro[a], **Libia Santos-Requejo**[b]

[a b] Universidad de Salamanca, Instituto de Estudios de la Ciencia y la Tecnología, Edificio I+D+i C/Espejo, 2, 37007, Salamanca

Contents

Appendix A: Eurobarometer questions 2

Appendix B: Reduction of within-country variance derived from each Science and Technology Studies model contribution 7

Appendix C: Reduction of within-country variance derived from each individual variable contribution 11

Appendix D: Countries by type of regime (Economist Democracy Index) 16

Appendix A: Eurobarometer questions

Text of the Eurobarometer 95.2 (2021) questions used to elaborate the variables of the document. Items here are presented in the same order as the variables are described in the main document.

CONSPI

Question Q20: For each of the following statements, please indicate whether you believe them to be true or false. If you don't know, you can indicate so. (READ OUT – ROTATE - ONE ANSWER PER LINE)

Number of item	Text of item	Answer options		
		True	False	DK
10	The cure for cancer exists but is hidden from the public by commercial interests (N)			
11	Viruses have been produced in government laboratories to control our freedom (N)			

SCLITERACY

Question Q20: For each of the following statements, please indicate whether you believe them to be true or false. If you don't know, you can indicate so. (READ OUT – ROTATE - ONE ANSWER PER LINE)

Number of item	Text of item	Answer options		
		True	False	DK
1	The earliest humans lived at the same time as the dinosaurs			
2	The continents on which we live have been moving for millions of years and will continue to move in the future			
3	Antibiotics kill viruses as well as bacteria			
4	The oxygen we breathe comes from plants			
5	Lasers work by focusing sound waves			
6	The world's human population is currently more than 10 billion (N)			
7	The methods used by the natural sciences and the social sciences are equally scientific (N)			
8	Human beings, as we know them today, developed from earlier species of animals (N)			
9	Climate change is for the most part caused by natural cycles rather than human activities (N)			

MISTRUST

Question Q10: The following are some statements that people have made about science and technology. For each statement, please indicate to what extent you agree or disagree. (READ OUT – ROTATE - ONE ANSWER PER LINE)

Number of item	Text of item	Answer options

		Totally agree	Tend to agree	Neither agree nor disagree	Tend to disagree	Totally disagree	DK
9	Because of their knowledge, scientists have a power that makes them dangerous						

Question Q11: To what extent do you agree with the following statements regarding scientists today? (READ OUT – ROTATE - ONE ANSWER PER LINE)

Number of item	Text of item	Answer options					
		Totally agree	Tend to agree	Neither agree nor disagree	Tend to disagree	Totally disagree	DK
1	We can no longer trust scientists to tell the truth about controversial scientific and technological issues because they depend more and more on money from industry						
2	Scientists only look at very specific issues and do not consider problems from a wider perspective						

NOTE: *In the data provided by the European Commission (2022), when the degree of agreement with a statement is requested, the respondent's answer is collected from the sequence 1 "Totally agree"; 2 "Tend to agree"; 3 "Neither agree nor disagree"; 4 "Tend to disagree"; 5 "Totally disagree". In our analysis, this sequence has been inverted so that a higher value indicates a greater quantity of the concept reflected by the item or indicator.*

INSTRUMENTAL

Question Q10: The following are some statements that people have made about science and technology. For each statement, please indicate to what extent you agree or disagree. (READ OUT – ROTATE - ONE ANSWER PER LINE)

Number of item	Text of item	Answer options					
		Totally agree	Tend to agree	Neither agree nor disagree	Tend to disagree	Totally disagree	DK
2	Science prepares the younger generation to act as well-informed citizens						
3	Thanks to scientific and technological advances, the Earth's natural resources will be inexhaustible						
4	Thanks to science and technology, there will be more opportunities for future generations						

CRITICAL

Question Q10: The following are some statements that people have made about science and technology. For each statement, please indicate to what extent you agree or disagree. (READ OUT – ROTATE - ONE ANSWER PER LINE)

Number of item	Text of item	Answer options					
		Totally agree	Tend to agree	Neither agree nor disagree	Tend to disagree	Totally disagree	DK
6	We depend too much on science and not enough on faith						
7	The applications of science and technology can threaten human rights						
8	Science makes our ways of life change too fast						

BLINDFAITH

Question Q9: The following are some statements that people have made about science or technology. For each statement, please indicate to what extent you agree or disagree. (READ OUT – ROTATE - ONE ANSWER PER LINE)

Number of item	Text of item	Answer options					
		Totally agree	Tend to agree	Neither agree nor disagree	Tend to disagree	Totally disagree	DK
7	Science and technology can sort out any problem						
8	There should be no limit to what science is allowed to investigate						
9	New inventions will always be found to counteract any harmful consequences of scientific and technological development						

ENGAGE

Question Q14: And now, a few questions on how you engage with science and technology issues. Do you: (READ OUT – ROTATE - ONE ANSWER PER LINE)

Number of item	Text of item	Answer options				
		Yes, regularly	Yes, occasionally	Hardly ever	No, never	DK
1	Talk about science and technology-related issues with family or friends					
2	Watch documentaries, or read science and technology-related publications, magazines or books					
3	Visit science and technology museums					
4	Study science and technology-related issues in your free time, for instance in a face-to-face or online course					
5	Sign petitions or join demonstrations on science and technology matters such as nuclear power, biotechnology, the environment or climate change					
6	Attend public meetings or debates about science and technology					
7	Take part in the activities of a non-governmental organisation dealing with science and technology related issues					

8	Contact public authorities or political leaders about science and technology-related issues					
9	Provide personal data for scientific research					
10	Take part in clinical trials					
11	Lend your computer's processing power to contribute to research on complex scientific questions					
12	Actively take part in scientific projects by developing research questions, collecting data, discussing the findings with others, etc.					

INVOLVEM

Question Q7: What level of public involvement do you think is appropriate when it comes to decisions about science and technology? (READ OUT — ONE ANSWER ONLY)

- The public does not need to be involved in decisions about science and technology (1)
- Decisions about science and technology should be made by scientists, engineers and politicians, but the public should always be informed (2)
- The public should be consulted and public opinion should be seriously considered when making decisions about science and technology (3)
- Public opinion should be the main concern when making decisions about science and technology (4)
- Other (SPONTANEOUS) (5)
- DK (6)

SEX

Question D10:

- Man (1)
- Woman (2)
- None of the above / Non binary / Do not recognize yourself in above categories (3)

NOTE: Only 44 respondents (0.1%) indicated option 3. The relative size of this group compared to the other two makes its inclusion in the analysis not very recommendable.

AGE

Question SD5:

How old are you?

ECONPREC

Question D60: During the last twelve months, would you say you had difficulties to pay your bills at the end of the month…?

- Most of the time (1)
- From time to time (2)
- Almost never/never (3)
- Refusal

Appendix B: Reduction of within-country variance derived from each Science and Technology Studies model contribution

Reduction of within-country variance derived from the contribution of the three models of the Social Studies of Science and Technology (SSST): Literacy, Public Understanding and Science in Society.

A) MODEL WITH SCLITERACY

Information Criteria[a]

-2 Log Likelihood	125110,58416963
Akaike's Information Criterion (AIC)	125118,58416963
Hurvich and Tsai's Criterion (AICC)	125118,58524855
Bozdogan's Criterion (CAIC)	125156,66739383
Schwarz's Bayesian Criterion (BIC)	125152,66739383

The information criteria are displayed in smaller-is-better form.
a. Dependent Variable: CONSPI.

Estimates of Fixed Effects[a]

						95% Confidence Interval	
Parameter	Estimate	Std. Error	df	t	Sig.	Lower Bound	Upper Bound
Intercept	1,616	,121	38,000	13,302	<,001	1,370	1,862
SCLITERACY_cmc	-,177	,004	37041,000	-47,890	,000	-,185	-,170

a. Dependent Variable: CONSPI.

Estimates of Covariance Parameters[a]

						95% Confidence Interval	
Parameter		Estimate	Std. Error	Wald Z	Sig.	Lower Bound	Upper Bound
Residual		1,700	,012	136,090	,000	1,675	1,724
Intercept [subject = NATION]	Variance	,559	,129	4,345	<,001	,356	,878

a. Dependent Variable: CONSPI.

(-2 Log L(EMPTY MODEL)) – (-2 Log L(SCLITERACY MODEL)) = 127335,87 - 125110,58 = 2225.29
Reduction of intra-nation variance = (1.805 – 1.700)/1.805 =5.8%

B) MODEL WITH PUBLIC UNDERSTANDING COMPONENTS

Model Dimension[a]

		Number of Levels	Covariance Structure	Number of Parameters	Subject Variables
Fixed Effects	Intercept	1		1	
	MISTRUST_cmc	1		1	
	INSTRUMENTAL_cmc	1		1	
	CRITICAL_cmc	1		1	
	BLINDFAITH_cmc	1		1	
Random Effects	Intercept	1	Identity	1	NATION
Residual				1	
Total		6		7	

a. Dependent Variable: CONSPI.

Information Criteria[a]

-2 Log Likelihood	121726,11388968
Akaike's Information Criterion (AIC)	121740,11388968
Hurvich and Tsai's Criterion (AICC)	121740,11695104
Bozdogan's Criterion (CAIC)	121806,66717539
Schwarz's Bayesian Criterion (BIC)	121799,66717539

The information criteria are displayed in smaller-is-better form.
a. Dependent Variable: CONSPI.

Estimates of Fixed Effects[a]

Parameter	Estimate	Std. Error	df	t	Sig.	95% Confidence Interval Lower Bound	95% Confidence Interval Upper Bound
Intercept	1,615	,121	38,000	13,304	<,001	1,369	1,861
MISTRUST_cmc	,387	,009	36555,012	40,920	,000	,369	,406
INSTRUMENTAL_cmc	-,123	,010	36555,008	-12,249	<,001	-,143	-,104
CRITICAL_cmc	,188	,010	36555,008	19,399	<,001	,169	,207
BLINDFAITH_cmc	,089	,009	36555,005	9,727	<,001	,071	,107

a. Dependent Variable: CONSPI.

Estimates of Covariance Parameters[a]

Parameter		Estimate	Std. Error	Wald Z	Sig.	95% Confidence Interval Lower Bound	95% Confidence Interval Upper Bound
Residual		1,620	,012	135,194	,000	1,597	1,644
Intercept [subject = NATION]	Variance	,558	,128	4,345	<,001	,356	,876

a. Dependent Variable: CONSPI.

(-2 Log L(EMPTY MODEL)) – (-2 Log L(PUBLIC UNDERSTANDING) = 127335,87 – 121726,11 = 5609.76
Reduction of intra-nation variance = (1.805 – 1.795)/1.805 = 10.2%

C) MODEL WITH SCIENCE IN SOCIETY COMPONENTS

Model Dimension[a]

		Number of Levels	Covariance Structure	Number of Parameters	Subject Variables
Fixed Effects	Intercept	1		1	
	ENGAGE_cmc	1		1	
	INVOLVEM_cmc	1		1	
Random Effects	Intercept	1	Identity	1	NATION
Residual				1	
Total		4		5	

a. Dependent Variable: CONSPI.

Information Criteria[a]

-2 Log Likelihood	125721,80524404
Akaike's Information Criterion (AIC)	125731,80524404
Hurvich and Tsai's Criterion (AICC)	125731,80687932
Bozdogan's Criterion (CAIC)	125779,35749547
Schwarz's Bayesian Criterion (BIC)	125774,35749547

The information criteria are displayed in smaller-is-better form.
a. Dependent Variable: CONSPI.

Estimates of Fixed Effects[a]

Parameter	Estimate	Std. Error	df	t	Sig.	95% Confidence Interval Lower Bound	95% Confidence Interval Upper Bound
Intercept	1,614	,121	38,000	13,288	<,001	1,368	1,860
ENGAGE_cmc	-,018	,002	36659,008	-11,242	<,001	-,021	-,015

INVOLVEM_cmc	,165	,010	36659,002	16,709	<,001	,145	,184

a. Dependent Variable: CONSPI.

Estimates of Covariance Parameters[a]

Parameter		Estimate	Std. Error	Wald Z	Sig.	95% Confidence Interval Lower Bound	Upper Bound
Residual		1,790	,013	135,386	,000	1,764	1,816
Intercept [subject = NATION]	Variance	,559	,129	4,344	<,001	,356	,877

a. Dependent Variable: CONSPI.

(-2 Log L(EMPTY MODEL)) – (-2 Log L(PUBLIC UNDERSTANDING) = 127335,87 – 125721,81 = 1614.06
Reduction of intra-nation variance = (1.805 – 1.795)/1.805 = 0.8%

Appendix C: Reduction of within-country variance derived from each individual variable contribution

Reduction of the variance within countries derived from the contribution of each individual variable of the Social Studies of Science and Technology included in the model.

A) EMPTY MODEL (RESULTS FOR COMPARISON)

Information Criteria[a]

-2 Log Likelihood	127335,86568961
Akaike's Information Criterion (AIC)	127341,86568961
Hurvich and Tsai's Criterion (AICC)	127341,86633694
Bozdogan's Criterion (CAIC)	127370,42810776
Schwarz's Bayesian Criterion (BIC)	127367,42810776

The information criteria are displayed in smaller-is-better form.
a. Dependent Variable: CONSPI.

Estimates of Fixed Effects[a]

Parameter	Estimate	Std. Error	df	t	Sig.	95% Confidence Interval Lower Bound	95% Confidence Interval Upper Bound
Intercept	1,616	,121	38,000	13,302	<,001	1,370	1,862

a. Dependent Variable: CONSPI.

Estimates of Covariance Parameters[a]

Parameter		Estimate	Std. Error	Wald Z	Sig.	95% Confidence Interval Lower Bound	95% Confidence Interval Upper Bound
Residual		1,805	,013	136,090	,000	1,779	1,831
Intercept [subject = NATION]	Variance	,559	,129	4,344	<,001	,356	,878

a. Dependent Variable: CONSPI.

B) MODEL WITH SCLITERACY

The reduction of variance produced by the SCLITERACY variable is the one that corresponds to the Science Literacy model, reflected in Section 2 (A) of this document, that is: 5.8%.

C) MODEL WITH MISTRUST

Information Criteria[a]

-2 Log Likelihood	123036,00741215
Akaike's Information Criterion (AIC)	123044,00741215
Hurvich and Tsai's Criterion (AICC)	123044,00849822
Bozdogan's Criterion (CAIC)	123082,06422719
Schwarz's Bayesian Criterion (BIC)	123078,06422719

The information criteria are displayed in smaller-is-better form.
a. Dependent Variable: CONSPI.

Estimates of Fixed Effects[a]

Parameter	Estimate	Std. Error	df	t	Sig.	95% Confidence Interval Lower Bound	95% Confidence Interval Upper Bound
Intercept	1,616	,122	38,000	13,289	<,001	1,370	1,863
MISTRUST_cmc	,486	,008	36797,000	61,300	,000	,471	,502

a. Dependent Variable: CONSPI.

Estimates of Covariance Parameters[a]

Parameter		Estimate	Std. Error	Wald Z	Sig.	95% Confidence Interval Lower Bound	95% Confidence Interval Upper Bound
Residual		1,643	,012	135,641	,000	1,619	1,667
Intercept [subject = NATION]	Variance	,560	,129	4,345	<,001	,357	,880

a. Dependent Variable: CONSPI.

(-2 Log L(EMPTY MODEL)) – (-2 Log L(MISTRUST MODEL)) = 127335,87 - 123036,01= 4299.86
Reduction of intra-nation variance = (1.805 – 1.643)/1.805 =9.0%

D) MODEL WITH INSTRUMENTAL

Information Criteria[a]

-2 Log Likelihood	126537,95550528
Akaike's Information Criterion (AIC)	126545,95550528
Hurvich and Tsai's Criterion (AICC)	126545,95659088
Bozdogan's Criterion (CAIC)	126584,01405742
Schwarz's Bayesian Criterion (BIC)	126580,01405742

The information criteria are displayed in smaller-is-better form.
a. Dependent Variable: CONSPI.

Estimates of Fixed Effects[a]

Parameter	Estimate	Std. Error	df	t	Sig.	95% Confidence Interval Lower Bound	95% Confidence Interval Upper Bound
Intercept	1,615	,121	38,000	13,304	<,001	1,369	1,861
INSTRUMENTAL_cmc	-,095	,009	36813,000	-10,439	<,001	-,113	-,077

a. Dependent Variable: CONSPI.

Estimates of Covariance Parameters[a]

Parameter		Estimate	Std. Error	Wald Z	Sig.	95% Confidence Interval Lower Bound	Upper Bound
Residual		1,804	,013	135,671	,000	1,778	1,830
Intercept [subject = NATION]	Variance	,558	,128	4,343	<,001	,355	,876

a. Dependent Variable: CONSPI.

(-2 Log L(EMPTY MODEL)) – (-2 Log L(INSTRUMENTAL MODEL)) = 127335,87 - 126537,96= 797.91
Reduction of intra-nation variance = (1.805 – 1.804)/1.805 =0.06%

E) MODEL WITH CRITICAL

Information Criteria[a]

-2 Log Likelihood	124433,00087935
Akaike's Information Criterion (AIC)	124441,00087935
Hurvich and Tsai's Criterion (AICC)	124441,00196454
Bozdogan's Criterion (CAIC)	124479,06095083
Schwarz's Bayesian Criterion (BIC)	124475,06095083

The information criteria are displayed in smaller-is-better form.
a. Dependent Variable: CONSPI.

Estimates of Fixed Effects[a]

Parameter	Estimate	Std. Error	df	t	Sig.	95% Confidence Interval Lower Bound	Upper Bound
Intercept	1,615	,121	38,000	13,302	<,001	1,369	1,861
CRITICAL_cmc	,399	,008	36827,000	48,157	,000	,383	,415

a. Dependent Variable: CONSPI.

Estimates of Covariance Parameters[a]

Parameter		Estimate	Std. Error	Wald Z	Sig.	95% Confidence Interval Lower Bound	Upper Bound
Residual		1,702	,013	135,696	,000	1,677	1,726
Intercept [subject = NATION]	Variance	,558	,129	4,344	<,001	,356	,877

a. Dependent Variable: CONSPI.

(-2 Log L(EMPTY MODEL)) – (-2 Log L(CRITICAL MODEL)) = 127335,87 – 124433.00= 2902.87
Reduction of intra-nation variance = (1.805 – 1.702)/1.805 = 5.71%

F) MODEL WITH BLINDFAITH

Information Criteria[a]

-2 Log Likelihood	126512,51738278
Akaike's Information Criterion (AIC)	126520,51738278
Hurvich and Tsai's Criterion (AICC)	126520,51846958
Bozdogan's Criterion (CAIC)	126558,57148208
Schwarz's Bayesian Criterion (BIC)	126554,57148208

The information criteria are displayed in smaller-is-better form.
a. Dependent Variable: CONSPI.

Estimates of Fixed Effects[a]

Parameter	Estimate	Std. Error	df	t	Sig.	95% Confidence Interval Lower Bound	95% Confidence Interval Upper Bound
Intercept	1,615	,121	38,000	13,306	<,001	1,370	1,861
BLINDFAITH_cmc	,017	,008	36772,000	2,004	,045	,000	,033

a. Dependent Variable: CONSPI.

Estimates of Covariance Parameters[a]

Parameter		Estimate	Std. Error	Wald Z	Sig.	95% Confidence Interval Lower Bound	95% Confidence Interval Upper Bound
Residual		1,810	,013	135,595	,000	1,784	1,836
Intercept [subject = NATION]	Variance	,558	,128	4,343	<,001	,355	,876

a. Dependent Variable: CONSPI.

(-2 Log L(EMPTY MODEL)) – (-2 Log L(BLINDFAITH MODEL)) = 127335,87 – 126512.52= 823.35
Reduction of intra-nation variance = (1.805 – 1.810)/1.805 = -0.28%

G) MODEL WITH ENGAGE

Information Criteria[a]

-2 Log Likelihood	126879,35593349
Akaike's Information Criterion (AIC)	126887,35593349
Hurvich and Tsai's Criterion (AICC)	126887,35701542
Bozdogan's Criterion (CAIC)	126925,42803082
Schwarz's Bayesian Criterion (BIC)	126921,42803082

The information criteria are displayed in smaller-is-better form.
a. Dependent Variable: CONSPI.

Estimates of Fixed Effects[a]

Parameter	Estimate	Std. Error	df	t	Sig.	95% Confidence Interval Lower Bound	95% Confidence Interval Upper Bound
Intercept	1,615	,121	38,000	13,307	<,001	1,369	1,860
ENGAGE_cmc	-,018	,002	36938,000	-11,261	<,001	-,021	-,015

a. Dependent Variable: CONSPI.

Estimates of Covariance Parameters[a]

Parameter		Estimate	Std. Error	Wald Z	Sig.	95% Confidence Interval Lower Bound	95% Confidence Interval Upper Bound
Residual		1,800	,013	135,901	,000	1,774	1,826
Intercept [subject = NATION]	Variance	,558	,128	4,344	<,001	,355	,875

a. Dependent Variable: CONSPI.

(-2 Log L(EMPTY MODEL)) – (-2 Log L(ENGAGE MODEL)) = 127335,87 – 126879,36= 456.51
Reduction of intra-nation variance = (1.805 – 1.800)/1.805 = 0.28%

H) MODEL WITH INVOLVEM

Information Criteria[a]

-2 Log Likelihood	126098,10292063
Akaike's Information Criterion (AIC)	126106,10292063
Hurvich and Tsai's Criterion (AICC)	126106,10400842
Bozdogan's Criterion (CAIC)	126144,15343235
Schwarz's Bayesian Criterion (BIC)	126140,15343235

The information criteria are displayed in smaller-is-better form.
a. Dependent Variable: CONSPI.

Estimates of Fixed Effects[a]

Parameter	Estimate	Std. Error	df	t	Sig.	95% Confidence Interval Lower Bound	95% Confidence Interval Upper Bound
Intercept	1,614	,121	38,000	13,286	<,001	1,368	1,860
INVOLVEM_cmc	,166	,010	36739,000	16,825	<,001	,146	,185

a. Dependent Variable: CONSPI.

Estimates of Covariance Parameters[a]

Parameter		Estimate	Std. Error	Wald Z	Sig.	95% Confidence Interval Lower Bound	95% Confidence Interval Upper Bound
Residual		1,795	,013	135,534	,000	1,769	1,821
Intercept [subject = NATION]	Variance	,559	,129	4,344	<,001	,356	,878

a. Dependent Variable: CONSPI.

(-2 Log L(EMPTY MODEL)) – (-2 Log L(INVOLVEM MODEL)) = 127335,87 – 126098,10 = 1237.77
Reduction of intra-nation variance = (1.805 – 1.795)/1.805 = 0.55%

Appendix D: Countries by type of regime (Economist Democracy Index)

Types of regimes			
**< 4**<br>**Authoritarian regime**	**4 to 5.99**<br>**Hybrid regime**	**6 to 7.99**<br>**Flawed democracy**	**>= 8**<br>**Full democracy**
Hungary	Poland	France	Belgium
Turkey	Slovenia	Italy	The Netherlands
Macedonia/FYROM	Bulgaria	Luxembourg	GERMANY
Montenegro	Kosovo	United Kingdom	Denmark
Serbia		Greece	Ireland
Albania		Spain	Portugal
Bosnia and Herzegovina		Austria	Finland
		Cyprus (Republic)	Sweden
		Czech Republic	Estonia
		Latvia	Norway
		Lithuania	Switzerland
		Malta	
