# Supplementary material for: Belief in science-related conspiracy theories is not just a matter of knowledge: The democratic quality of countries as a protective factor
Source: PLoS One. 2025 Dec 18;20(12):e0338384. doi: 10.1371/journal.pone.0338384 (PMC12714214; doi:10.1371/journal.pone.0338384)
Supplement: Attachment — Submitted filename: Review PONE-D-25-29874.docx [file pone.0338384.s002.docx]

This research article investigates which factors are associated with belief in science-related conspiracy theories (SCTs) across Europe. Using Eurobarometer survey data from over 37,000 participants, the authors examine how scientific literacy, attitudes toward science, and the democratic quality of a country relate to SCT beliefs at both individual and national levels.

The primary aim is to test three established models of public understanding of science: Science Literacy, Public Understanding, and Science-in-Society by analyzing their links with SCT belief. In addition, the study asks whether a country’s democratic quality shapes these associations. The authors use cross-sectional survey data and multi-level regression modeling to explore these questions.

**Main Hypotheses and Key Findings**

- H1: People with higher scientific knowledge are less likely to believe in science-related conspiracy theories (SCTs).

Finding: The data support this. The research showed a negative and significant relationship between scientific literacy (SCLITERACY) and belief in SCTs. However, this link is much stronger in countries with higher democracy. In authoritarian or hybrid regimes, an increase in scientific knowledge leads to only a minimal reduction in conspiracy beliefs. In full democracies, scientific knowledge makes a real difference, sometimes nearly eliminating SCT belief.

- H2: People with a more positive attitude toward science are less likely to believe in SCTs.
  H2.1: Seeing more benefits in science predicts lower SCT belief.
  H2.2: Trusting scientists predicts lower SCT belief.

Finding: These are confirmed, especially for trust. Mistrust in scientists is the most potent driver of conspiracy belief, much more than simply lacking knowledge. Incorporating these attitudinal variables, the "Public Understanding" model explained the most significant proportion of intra-country variance (10.2%), highlighting its strong explanatory power. This suggests that the rise of these theories is deeply connected to a strained relationship with scientific authority and growing institutional distrust. The study also found that individuals who were more prone to identifying the potential harms of science and technology (CRITICAL view) were more likely to believe in SCTs. Surprisingly, those with an overly optimistic or "blind faith" view of science (BLINDFAITH) also showed a higher tendency to believe in SCTs.

- H3: People more involved with science will believe less in SCTs.
  H3.1: Taking part in science-related activities is linked to lower SCT beliefs.
  H3.2: Favoring public involvement in science decision-making should predict lower SCT belief.

Finding: There is partial support. Being active in science (like following popular science or participating in citizen science) is linked to less belief in conspiracy theories. But surprisingly, people who want more lay involvement in science policy have higher conspiracy beliefs.

- H4: People in less democratic countries are more likely to believe in SCTs.

Finding: This is strongly supported. Living in a democracy lowers the odds of believing in science-related conspiracies. Once a country passes a certain threshold for democracy, SCT belief drops sharply.

- H5: The relationship between attitudes/knowledge about science and conspiracy belief is influenced by how democratic a country is.

Finding: The moderating effect of democracy is only observed in the case of scientific knowledge. Democracy strengthens the negative link between knowledge and conspiracy belief, making knowledge more “protective” in democratic countries. For other attitudes (like trust), this interaction is not significant.

**Other Findings (Control Variables):**

- Age: Younger people are more likely to believe in SCTs.
- Economic hardship: Financial difficulties are linked to greater conspiracy belief.
- Gender: No significant effect on this dataset.

In short, this study shows that belief in science-related conspiracy theories cannot be explained by lack of knowledge alone. The most critical factors are trust in scientists and the broader democratic context. Living in a democratic society reduces SCT belief, even among those with less knowledge or higher mistrust. The findings strongly suggest that public policies aimed at combating misinformation should be tailored to specific national contexts, rather than relying on a one-size-fits-all approach that primarily emphasizes knowledge dissemination.

Given the study's strengths, especially its robust methodology, the breadth of its dataset, and its thoughtful analytical approach, I believe this manuscript makes a valuable contribution to the literature on science-related conspiracy beliefs. With a few clarifications and minor improvements as suggested below, the article would be well-suited for publication in PLOS ONE. Addressing these points will help further strengthen the clarity and practical value of the paper.

**Key Strengths:**

1- Addressing a Critical and Timely Issue:

This paper addresses the significant issue of conspiracy theories related to science, which have substantial implications for both individual and public health. The research feels particularly relevant given today’s climate of misinformation and growing public concern about the impact of these beliefs.

2- Having a Robust Methodology and a Large Dataset:

The paper uses data from a Special Eurobarometer survey, with 37,079 participants drawn from 27 EU countries and 11 other countries or territories. This large and diverse sample makes the findings generalizable and statistically solid. Multilevel Linear Modelling (MLM) fits the nested structure of the data, individuals within countries, and lets the authors analyze individual and national factors and their interactions. The ICC of 0.236 shows that nearly a quarter of the variation in conspiracy beliefs comes from country-level differences, which confirms that MLM is appropriate.

3- Challenging the “Deficit Model” and Offering a Nuanced Perspective:

One of the article’s central contributions is challenging the standard idea that believing in conspiracy theories is solely a lack of knowledge. The authors show that, although scientific literacy is linked to lower conspiracy beliefs, mistrust in scientists is a more decisive factor. They also highlight the protective effect of democracy at the national level, moving beyond the traditional “deficit model”.

4- Integrating Established Theoretical Frameworks:

The research paper is notable for systematically testing three major models from literature: Science Literacy, Public Understanding, and Science-in-Society. This comprehensive approach gives a nuanced picture of why people accept or reject science-related conspiracy theories.

5- Offering Innovative Focus on Country-Level Factors:

Another key strength is the study examining how democracy moderates the relationship between science perceptions and conspiracy beliefs. This focus on national context is original. The findings show that democracy acts as a buffer: people in more democratic countries are less likely to believe in SCTs, even when their trust in scientists is low or their scientific knowledge is limited.

6- Highlighting Practical Policy Implications:

The results are directly relevant for policymakers. The authors show that effective responses to misinformation can’t be one-size-fits-all. Policy needs to be tailored to each country’s context, not just focused on increasing knowledge.

7- Identifying Key Factors:

Finally, the study indicates that mistrust of scientists is the primary factor driving belief in science-related conspiracy theories, which account for the most significant proportion of within-country variation.

**Areas for Improvement**

1- Partial Confirmation of Hypotheses

1.1 Hypothesis 3 (Engagement in Science): This hypothesis received only partial support. Engagement in scientific dissemination activities (ENGAGE) showed the expected negative correlation with SCT belief, but, interestingly, a favorable attitude towards citizen involvement in science decision-making (INVOLVEM) was linked to higher belief in SCTs.

*Suggested improvement:* Expanding the discussion around this unexpected result would be beneficial. The authors interpret this pattern as reflecting “alternative epistemologies” and dissatisfaction with academic elites. However, further theoretical exploration and/or qualitative research could clarify why greater support for lay involvement is associated with increased conspiracy belief. The authors may consider strengthening this part of the discussion by referencing the growing literature on science populism and anti-expert sentiment to clarify whether this association reflects a genuine demand for participatory democracy or, rather, a reaction against elites. It would be beneficial to cite recent studies and deepen the interpretation, enriching the discussion and situating the findings within broader debates.

In addition, recent scholarship shows a growing debate on the link between conspiracy beliefs and civic or political engagement. While some studies, such as Jolley and Douglas (2014) argue that conspiracy beliefs may suppress participation, others, like Kim (2022), find that conspiracy beliefs can stimulate engagement. In this paper, the unexpected positive association between support for citizen involvement in science decision-making and SCT belief (H3) could be better supported by directly referencing these contrasting perspectives in the literature. This would help readers understand such findings. However, at first counterintuitive, it aligns with emerging evidence that conspiracy beliefs sometimes motivate individuals to seek greater influence and involvement, often as a reaction to perceived exclusion by experts or institutions.

1.2 Hypothesis 5 (Moderation Effect): This hypothesis was only partially supported. The moderating effect of democracy was apparent in the relationship between scientific knowledge and SCT belief, but not in the link between distrust of scientists and conspiracy belief, despite distrust being a strong individual-level predictor.

*Suggested improvement:* It would be beneficial to expand the discussion on why democracy moderates the effect of knowledge, but not of distrust, especially since mistrust is such a central predictor. This could expand the theoretical contribution of the manuscript.

2- Nuance in Attitudinal Variables

The result that “blind faith in science” (BLINDFAITH) positively correlates with SCT belief is interesting. The authors interpret this as a difference between trust in scientific methods and distrust in those who carry them out (the “academic elite”).

*Suggested improvement*: It would be beneficial to elaborate further on how “trust” or “faith” in science can be more precisely measured, or how naive faith manifests behaviorally among conspiracy believers. Exploring these facets in future research could deepen the field’s understanding.

.

3- Lower Explanatory Power of the “Science-in-Society” Model

The “Science-in-Society” model, which emphasizes engagement, accounted for less intra-country variance (0.8%) compared to the “Public Understanding” (10.2%) and “Science Literacy” (5.8%) models.

*Suggested improvement:* While not a weakness of the study itself (as it accurately reports the findings), It would be beneficial to offer a brief discussion on why this model, which often emphasizes public participation, might have less direct explanatory power for conspiracy belief in this context, or under what conditions its influence might be more substantial.

4- Interpretation of Democracy versus Other Country-Level Predictors:

The main result is that democracy at the country level is negatively associated with belief in science-related conspiracy theories. However, democracy is highly correlated with other macro-level factors such as GDP, corruption, and government effectiveness. This collinearity means that the effect attributed to democracy may not be truly independent. The authors mention this, but it would be beneficial to make this caution even more explicit, especially in the interpretation and implications sections. The authors may consider noting more clearly that the current models cannot fully separate the effect of democracy from these other macro factors.

5- Omission of Education as a Control

Education is a well-established predictor of conspiracy beliefs in prior research. In this paper, the authors use scientific knowledge ("Scientific Literacy") as their primary explanatory variable, rather than including general education as a control. This is a plausible decision since scientific literacy is closely related to formal education and directly aligns with the study’s theoretical framework. The authors position "Scientific Literacy" as a more targeted measure of the knowledge component under investigation.

However, since education is so commonly used as a control in literature, its omission limits the comparability of these results with previous studies. The authors may consider briefly clarifying their decision not to include education. A short discussion of this point may increase transparency and help readers situate the findings within the broader research field.

In conclusion, this article provides significant insights into the relationship between scientific literacy, trust in science, and the role of democracy in shaping belief in science-related conspiracy theories. While the paper is already strong in its design and execution, some targeted clarifications will make the contribution more transparent and accessible to a broad audience.

Jolley, D., & Douglas, K. M. (2014). The social consequences of conspiracism: Exposure to conspiracy theories decreases intentions to engage in politics and to reduce one’s carbon footprint. *British Journal of Psychology*, *105*(1), 35–56. https://doi.org/10.1111/bjop.12018

Kim, Y. (2022). How conspiracy theories can stimulate political engagement. *Journal of Elections, Public Opinion and Parties*, *32*(1), 1–21. https://doi.org/10.1080/17457289.2019.1651321
