# Supplementary material for: Belief in science-related conspiracy theories is not just a matter of knowledge: The democratic quality of countries as a protective factor
Source: PLoS One. 2025 Dec 18;20(12):e0338384. doi: 10.1371/journal.pone.0338384 (PMC12714214; doi:10.1371/journal.pone.0338384)
Supplement: Attachment — Submitted filename: Response Reviewer 3.docx [file pone.0338384.s003.docx]

Reviewer #3: Dear authors,

Thank you for giving me the opportunity to review your manuscript. I believe this is excellent research and only have a few comments.

Line 61: the first quotation mark does not have a closing quotation mark (")

We thank the reviewer for noticing this oversight. The missing closing quotation mark has been corrected in the revised version of the manuscript.

Line 63: academic elite's (missing apostrophe)

We appreciate the reviewer’s careful attention to detail. However, the phrase in question is part of a direct quotation from the original source. Therefore, we have kept the wording unchanged to preserve the accuracy of the cited text.

Line 63: sovereignty (misspelled)

We have carefully checked the manuscript, and the term *“sovereignty”* is correctly spelled in the revised version.

Line 246: the authors use the word "prove"; this is not a word that should be employed in empirical research as it is impossible to prove something with 100% certainty

We thank the reviewer for this important observation. We fully agree that the term *“prove”* conveys an inappropriate level of certainty in the context of empirical research. Accordingly, we have revised the sentence to adopt a more accurate formulation. The phrase now reads:

“Based on these previous indications, in our study we aim to **test whether** the quality of democracy in European countries moderates the relationship between individuals’ attitudes toward science and belief in SCT.”

This change ensures a more precise and methodologically appropriate description of our research objective.

Line 344: inconsistency in the numbering of hypotheses

We thank the reviewer for pointing out this inconsistency. The numbering has been corrected in the revised manuscript, and Hypothesis 2 now includes its two sub-hypotheses (H2.1 and H2.2) within the same paragraph for greater clarity and coherence.

Line 383: the equation is not clear (the parentheses are not appropriately sized)

The equation has been corrected in the revised version of the manuscript to ensure proper formatting.

Lines 452-455: the whole sentence is awkward

We agree that the sentence contained structural and stylistic issues, which have now been corrected in the revised version to improve clarity and readability.

Line 457: The authors refer to "works" but do not cite any sources

The sentence in question was indeed incomplete and lacked proper citation. However, this part has been removed in the revised version of the manuscript, as we have reformulated the interpretation of the attitudinal variables in the discussion section to improve clarity and coherence.

Note for the hypotheses: the authors present the hypotheses with little to no text referring to these hypotheses in the main text. Usually when we include numbered hypotheses, we need to refer to them directly in the text. The authors do so for hypothesis 3 but not for the others.

We thank the reviewer for this helpful observation. In the revised version, all hypotheses and sub-hypotheses are now explicitly mentioned and referenced in the Results section, ensuring consistency and clearer connection between the theoretical framework and the empirical analysis.

Apart from these minor comments, I think this manuscript looks great and will make a good contribution to the research agenda on conspiracy beliefs, particularly through the multilevel and cross-national approach.
