# Supplementary material for: Belief in science-related conspiracy theories is not just a matter of knowledge: The democratic quality of countries as a protective factor
Source: PLoS One. 2025 Dec 18;20(12):e0338384. doi: 10.1371/journal.pone.0338384 (PMC12714214; doi:10.1371/journal.pone.0338384)
Supplement: Attachment — Submitted filename: Plos one review.docx [file pone.0338384.s004.docx]

Reviewer Recommendation and Comments for the Author

Recommendation: Accept

General Assessment:

I acknowledge the authors' response to the initial review. The revisions provided in the rebuttal letter and the corresponding edits in the text address the concerns raised. The manuscript has been improved, particularly in terms of the theoretical framework and the interpretation of the statistical findings. The inclusion of new literature regarding science populism and the articulation of the distinction between institutional and affective dimensions of trust have clarified the paper’s contribution.

Below, I provide a detailed assessment of how the specific points have been addressed:

1. Hypothesis 3 (Engagement in Science) In the initial review, I noted the paradoxical finding regarding the positive correlation between support for citizen involvement in science decision-making and belief in science-related conspiracy theories. The authors have expanded the discussion (lines 440–477) to address this issue.

- Theoretical Integration: The integration of the "science populism" framework (referencing Mede & Schäfer; Merkley) and the inclusion of the debate on civic engagement (comparing Jolley & Douglas vs. Kim) provides the necessary theoretical context.
- Interpretation: Interpreting this finding as a tension between "epistemic distrust" and "participatory demands" offers a logical resolution. The manuscript now positions this result as evidence of "alternative epistemologies" where conspiracy believers seek to reclaim power from perceived elites. This revision clarifies the findings within the study's framework.

2. Hypothesis 5 (The Moderating Role of Democracy) The authors were asked to clarify why democracy moderates the effect of scientific knowledge but not the effect of distrust. The expanded discussion (lines 526–539) offers a plausible explanation.

- Distinction of Mechanisms: The authors argue that scientific knowledge is "context-dependent," shaped by educational quality, transparency, and institutional reliability—conditions that are generally stronger in consolidated democracies. By contrast, distrust in scientists is characterized as an "affective or identity-based orientation," which is less shaped by the institutional environment and more by cultural or populist narratives.
- Assessment: This theoretical distinction is sound. It explains why "affective distrust" persists in democratic contexts, providing a consistent explanation for the null finding regarding the moderation of distrust.

3. Blind Faith in Science (BLINDFAITH) The authors have addressed the comments regarding the conceptual limitations of the "Blind Faith" variable.

- Assessment: They have added a clarification acknowledging that the current variable captures a generalized confidence rather than a specific trust in methodology (lines 557–567). The outline of behavioral indicators for "naïve scientism" is a useful addition to the discussion for future research.

4. Low Explanatory Power of the Science-in-Society Model The revised text (lines 440–447) provides a clear explanation for the model’s lower explanatory power (0.8%).

- Assessment: Clarifying that Science-in-Society variables capture "normative beliefs" (how science *should* function) rather than beliefs about actual scientific practice is a valid interpretation. Acknowledging that these variables may influence SCT belief only indirectly and depend on national context places the findings in the correct perspective.

5. Democracy vs. Other Country-Level Predictors The authors have updated the discussion in several places (lines 496–500, 553–558) regarding the collinearity between democracy and other macro-factors.

- Assessment: The explicit statement that democracy is highly correlated with GDP, corruption, transparency, and governance indicators addresses the methodological concern. By acknowledging that the current model cannot fully isolate democracy’s independent causal effect from broader institutional environments, the interpretation is methodologically transparent.

6. Omission of Education as a Control Variable I accept the explanation provided in the methodology section (lines 229–235) for omitting education as a control variable.

- Assessment: The justification based on data quality issues in the Eurobarometer education items (including implausible values) and the potential multicollinearity with scientific literacy is valid. The decision to focus on scientific literacy is appropriate given the study's theoretical focus and data constraints.

Conclusion: The authors have addressed the theoretical and methodological points raised during the review process. The revised manuscript presents a multi-level analysis that is grounded in the relevant literature.
